# Improvement in Grip Strength Following Percutaneous Needle Aponeurotomy for Dupuytren’s Disease: A Prospective Clinical Study

**DOI:** 10.1177/15589447261416974

**Published:** 2026-02-21

**Authors:** Ishith Seth, Brett K. Sacks, Omar Shadid, Richard J. Ross, Warren M. Rozen

**Affiliations:** 1 Peninsula Health, Frankston, VIC, Australia; 2 Monash University, Frankston, VIC, Australia

**Keywords:** Dupuytren’s disease, grip strength, percutaneous needle aponeurotomy, hand function, joint contracture

## Abstract

**Background:**

Dupuytren’s disease causes progressive flexion contractures and impaired hand function. While percutaneous needle aponeurotomy (PNA) provides rapid correction with low morbidity, the effect on grip strength remains unclear. Grip strength is a key surrogate of hand function and overall health, yet it has been inconsistently evaluated as an outcome in Dupuytren’s disease.

**Methods:**

A prospective study was conducted at a tertiary referral center including 53 patients (80 digits) treated with PNA between February 2024 and March 2025. Standardized assessments included grip strength (Jamar dynamometer, American Society of Hand Therapists protocol), joint extension deficits, patient-reported outcomes (Southampton, Unité Rhumatologique des Affections de la Main [URAM]), and return-to-work data. Grip strength was reassessed at 2 months postprocedure to capture early functional recovery. Statistical analysis used paired *t* tests and Wilcoxon signed-rank tests, with significance set at *P* < .05.

**Results:**

Mean grip strength improved from 24.9 to 28.7 kg (mean change + 3.8 kg, *P* < .001). Extension deficits decreased significantly at all levels, with mean correction of 25.5° at the metacarpophalangeal joint (MCPJ) and 29.3° at the proximal interphalangeal joint (PIPJ) (*P* < .001). At 2 months, the median URAM score was 4 out of 45, and the Southampton score was 3 out of 20, reflecting excellent functional recovery. Forty-four employed patients returned to work within 1 week. Complications were minor (skin tears n = 9, transient hypersensitivity n = 1) with no major adverse events.

**Conclusions:**

Percutaneous needle aponeurotomy (PNA) not only corrects digital contracture but also yields clinically significant improvements in grip strength, reinforcing its value as a functional outcome measure. These findings support PNA as a safe, effective first-line treatment for selected patients with Dupuytren’s disease.

## Introduction

Dupuytren’s disease (DD) is a chronic, progressive fibroproliferative disorder of the palmar fascia that results in flexion contractures of the fingers and impaired hand function.^1^ Treatment options for DD have evolved significantly in recent decades. Historically, open surgical fasciectomy was the standard of care, particularly for patients with advanced diseases.^1,2^ While effective in achieving contracture release, open fasciectomy is associated with considerable morbidity, including wound healing complications, neurovascular injury, joint stiffness, and a prolonged recovery period. In contrast, minimally invasive techniques such as collagenase Clostridium histolyticum (CCH) injection and percutaneous needle aponeurotomy (PNA) have gained popularity for early to moderate disease due to their favorable safety profiles, lower cost, and shorter rehabilitation time.^1,3^ Percutaneous needle aponeurotomy, in particular, involves the percutaneous division of pathological cords using a hypodermic needle, allowing for immediate passive extension and functional recovery.^4^ Although recurrence rates may be higher compared with open procedures, the balance of efficacy, safety, and patient satisfaction has made PNA an attractive first-line intervention in selected patients.^4^

Grip strength is a widely used surrogate of global hand function and muscular integrity and is independently predictive of disability, morbidity, and even mortality in aging populations.^5^ However, its role in evaluating DD severity and treatment success remains unclear. Some studies have demonstrated that patients with DD generally retain near-normal maximal grip strength despite digital deformity.^6^ Compensatory mechanisms may explain this paradox, including the preservation of intrinsic and extrinsic hand musculature or the limited involvement of key gripping digits, such as the index and thumb.^6^ Nevertheless, some studies suggest that grip efficiency and fine motor coordination may be impaired in DD, particularly during precision tasks, even when gross strength measures appear preserved.^7^

Percutaneous needle aponeurotomy is associated with rapid return to activity, minimal soft tissue trauma, and high patient satisfaction.^1^ The current literature examining the effect of interventions for DD on grip strength is limited and yields inconsistent conclusions.^4,8,9^ Given that the procedure restores digital extension and potentially relieves mechanical constraints on the flexor apparatus, an improvement in grip strength may be anticipated following treatment. Yet this relationship remains speculative, with most previous studies focused primarily on contracture release and recurrence rates rather than functional performance metrics. Although PNA may contribute to improvement in grip strength through release of pathological cords and restoration of digital range, further high-quality studies are required to clarify whether such functional gains are consistent, sustained, and clinically meaningful across broader populations.

In light of these gaps, this prospective clinical study aimed to assess changes in grip strength following percutaneous needle aponeurotomy for DD. We hypothesized that patients would experience a measurable improvement in grip strength following successful correction of contracture deformities, particularly in moderate-to-severe cases. This study, therefore, aimed to objectively quantify changes in grip strength following PNA using standardized dynamometry.

## Methodology

A prospective clinical study was conducted at a single Australian tertiary referral hospital to evaluate changes in grip strength following PNA in patients diagnosed with DD. Ethics approval was obtained from the institutional review board (QA/106427/PH-2024-418696(v2)), and informed consent was collected from all participants in accordance with the 1964 Declaration of Helsinki.

### Participant Selection

Eligible participants were adults aged 18 years or older, with a clinical diagnosis of DD involving the metacarpophalangeal joint (MCPJ) and/or proximal interphalangeal joint (PIPJ), who were deemed suitable for PNA by the senior hand surgeon. Exclusion criteria included patients with coexisting hand conditions affecting grip strength (eg, symptomatic carpal tunnel syndrome, severe arthritis, or trigger finger) in the treated hand, as well as those with prior surgery or intervention within the last 6 months. Demographic information, including age, sex, occupation, dominant hand, comorbidities (including diabetes mellitus and ischemic heart disease), smoking status, alcohol use, affected hand and previous management of DD. In addition, baseline measurements of flexion contracture were recorded using a goniometer for all involved digits at the MCPJ and PIPJ.

### PNA Procedure

All procedures were performed under sterile conditions by a single experienced hand surgeon. The PNA technique involved percutaneous division of the fibrous cords using the bevel of a 21-gauge hypodermic needle after infiltration with 1% lidocaine. Cords were sectioned at multiple points, followed by passive extension of the affected fingers to rupture the pathologic tissue. Patients without diabetes received an intralesional injection of 1 mL triamcinolone acetonide (40 mg/mL) following cord rupture.

Postoperatively, all patients were reviewed by a hand therapist on the same day. A static extension splint was fabricated and worn nightly for 3 months. Patients were also educated on a range of motion exercises and encouraged to return to light daily activities as tolerated. Two additional hand therapy reviews were scheduled during the 2-month follow-up period.

### Outcome Measures

The primary outcome was grip strength, measured using a Jamar hydraulic hand dynamometer (model 5030 J1, Sammons Preston Rolyan, Bolingbrook, Illinois). Grip strength was assessed pre-PNA, and again at 2 months postprocedure. Measurements were recorded in kilograms-force (kgf). Follow-up assessments were performed at 2 months postprocedure to capture early functional recovery consistent with routine clinical review intervals at our institution. The dynamometer was set at the second handle position, and all measurements followed a standardized testing protocol recommended by the American Society of Hand Therapists. Each participant was seated with the shoulder adducted and neutrally rotated, the elbow flexed at 90°, the forearm in a neutral position, and the wrist in a 0° to 30° extension. Each participant was instructed to squeeze the dynamometer with maximal effort for 5 seconds during each trial. Three attempts were recorded for each hand, and the maximum value was used for analysis. The same blinded assessor performed all dynamometry to reduce inter-rater variability.

Secondary outcomes included changes in passive extension deficits at the MCP and PIP joints, recorded using a goniometer. Values were expressed in degrees, with hyperextension recorded as zero. Involvement of specific digits and Tubiana grading were documented at baseline. Patient-reported outcome measures, including the Southampton score (0-20) and the URAM (Unité Rhumatologique des Affections de la Main) score (0-45), were recorded at the 2-month follow-up. Lower scores indicated better functional outcomes. Time to return to work or unrestricted activity was also recorded. Complications, including skin tears, neurovascular injuries, infections, recurrences, and other adverse events, were systematically documented at each follow-up.

### Statistical Analysis

Data were analyzed using STATA version 17. Descriptive statistics were used to summarize baseline characteristics. Continuous variables were reported as means and standard deviations or medians and ranges, depending on data distribution. Paired *t* tests or Wilcoxon signed-rank tests were used to compare pre- and postprocedural grip strength and extension deficits, with a *P* value of <.05 considered statistically significant. Subgroup analyses were performed based on MCPJ and PIPJ contracture improvement.

## Results

Data were collected from March 2024 to March 2025. A total of 53 patients underwent PNA for Dupuytren’s contracture, encompassing 119 joint releases across 80 digits. Baseline demographic and clinical characteristics are summarized in Table 1. Grip strength, measured by Jamar dynamometer, increased significantly from a mean of 25 to 28.8 kg (mean change: + 3.8 kg, *SD* = 6.3; *P* < .001, Table 2).

**Table 1.** Baseline Characteristics of Study Participants.

Variables	Value
Total, N	53
Age (years), median (range)	66 (38-84)
Sex, n	
Men	41
Women	12
Medical comorbidities	
Type 2 diabetes mellitus	9
Previous Dupuytren’s intervention	21
Heart disease	6
Social history, n	
Smoker	19
Alcohol use (>15 g/day)	20
Affected hand, n	
Left	16
Right	37
Dominant hand	34
Digits involved, n	
Thumb	6
Index	2
Middle	14
Ring	29
Little	29
Affected joints, n	
MCPJ	61
PIPJ	51
DIPJ	7
Type of work	
Manual	44
Nonmanual	9

*Note.* MCPJ = metacarpophalangeal joint; PIPJ = proximal interphalangeal joint; DIPJ = distal interphalangeal joint.

**Table 2.** Joint Contracture (Extension Deficit in Degrees) and Grip Strength (kg) at Baseline and Day 60 Post-PNA.

Outcome measure	N	Mean baseline	Mean D 60	Mean change	*SD* change	Test	*P* value
MCPJ	47	31.27	5.74	−25.53	20.35	Wilcoxon	<.0001
PIPJ	44	36.47	7.15	−29.31	28.60	Wilcoxon	<.0001
DIPJ	5	20.00	0	−20.00	14.14	Wilcoxon	.05
Grip strength	53	24.95	28.75	+3.80	6.34	Wilcoxon	<.0001

*Note.* Joint extension deficits are presented for the MCPJ, PIPJ, and DIPJ, with grip strength measured in kilograms using a Jamar dynamometer. Data is presented as means with standard deviation and *P* values from Wilcoxon or paired *t* test comparisons. PNA = percutaneous needle aponeurotomy; SD = standard deviation; MCPJ = metacarpophalangeal joint; PIPJ = proximal interphalangeal joint; DIPJ = distal interphalangeal joint.

The median age of the participants was 66 years (range: 38-84), and the cohort consisted of 41 men and 12 women. A history of type 2 diabetes was reported in 9 patients, heart disease in 6, and 21 patients had previously undergone intervention for DD. Smoking history was documented in 19 patients (36%), and 20 patients (38%) reported moderate daily alcohol consumption. The right hand was affected in 37 patients, with left-sided involvement in 16, and 34 cases involved the dominant hand. The little and ring fingers were the most affected digits, each involved in 29 cases, followed by the middle finger (14), thumb (6), and index finger (2). Joint involvement was noted in the MCPJ in 61 cases, the PIPJ in 51 cases, and the distal interphalangeal joint (DIPJ) in 7 cases. Manual labor was the predominant occupational category, with 44 patients engaged in manual work and 9 in nonmanual roles. The majority of contractures involved single or dual-digit releases. No major complications were recorded. Minor skin tears occurred in 9 cases and resolved without infection. One case of postprocedure hypersensitivity was recorded, with symptoms affecting digits not involved in the procedure.

Improvement in joint contracture following PNA was significant (Table 2). Metacarpophalangeal joint (MCPJ) extension deficits improved from a mean of 31.3° at baseline to 5.7° at day 60 (mean change: −25.5°, *SD* = 20.4; *P* < .001). Similarly, PIPJ deficits improved from a mean of 36.5° to 7.2° (mean change: −29.3°, *SD* = 28.6; *P* < .001). D﻿istal interphalangeal joint (DIPJ) improvement was observed in a smaller subset (n = 5), with mean extension deficit reducing from 20° to 0° (−20.0°, *SD* = 14.1; *P* = .05).

**Figure 1.**
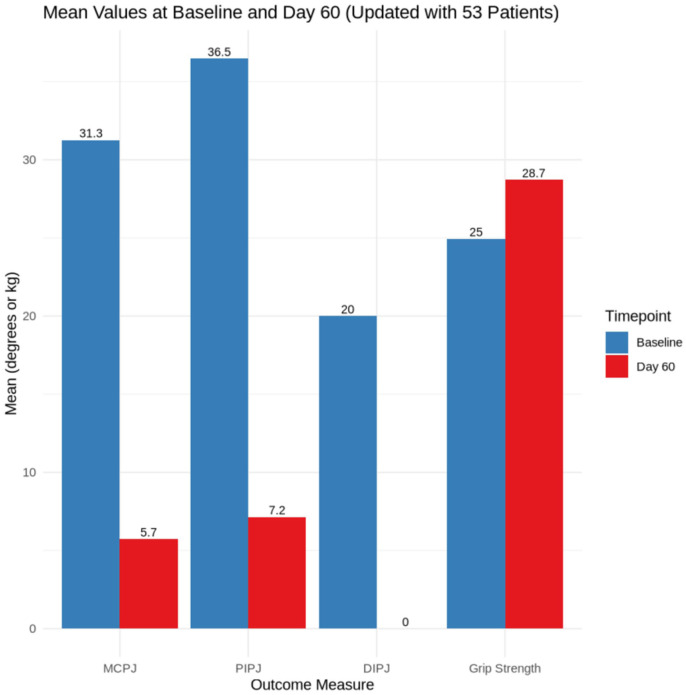
Mean values of outcome measures at baseline and day 60 following percutaneous needle aponeurotomy. *Note.* Joint extension deficits (in degrees) are presented for the MCPJ, PIPJ, and DIPJ, with grip strength measured in kilograms using a Jamar dynamometer. Bars represent mean values at each time point. A marked reduction in joint contracture and an increase in grip strength were observed by day 60. MCPJ = metacarpophalangeal joint; PIPJ = proximal interphalangeal joint; DIPJ = distal interphalangeal joint.

Figure 1 visually summarizes the improvements in joint contracture and grip strength following PNA. Consistent with the statistical analysis in Table 2, all outcome measures showed substantial functional improvement by day 60. Notably, the PIPJ exhibited the largest reduction in contracture, while gains in grip strength reflected the early restoration of functional capacity. This pattern underscores the effectiveness of PNA in both structural release and functional recovery across a diverse patient cohort.

All working participants (n = 44) returned to work within 1 week following the procedure, demonstrating early functional recovery and minimal downtime. The sole exception was one patient who experienced postprocedural hypersensitivity in uninvolved digits and delayed their return to work until 6 weeks after PNA. At the 2-month follow-up, patient-reported outcome measures indicated excellent subjective function and minimal residual disability. The median Southampton score was 3 out of 20 (range 0-14), and the median URAM score was 4 out of 45 (range 0-29). These low scores reflect minimal discomfort and very limited or no perceived impact of DD on daily activities and hand function after treatment.

## Discussion

This prospective clinical study demonstrates that percutaneous needle aponeurotomy results in significant improvements in both joint contractures and grip strength in individuals with DD. At 2 months postprocedure, patients achieved a mean increase in grip strength of 3.76 kg (*P* < .001). Although joint contracture correction is the primary target of DD management, few studies have focused on the relationship between structural correction and active functional recovery, such as grip strength. The mean reduction in MCPJ and PIPJ contractures by 25.5° and 29.3°, respectively, directly contributes to improved hand positioning and biomechanics, likely facilitating more effective gripping capacity. This aligns with prior reports that underscore the importance of finger extension in enabling the mechanical components of grip.^5,10,11^

The role of grip strength in DD has been inconsistently addressed, with earlier research often concluding that patients retain near-normal grip strength despite deformity.^10^ Prior studies have variably reported the effect of contracture release on grip strength. Although some demonstrated subjective or modest objective improvements.^12-14^ others found no change following collagenase injection.^14^ Our results align with those of Medland et al, who observed early gains after percutaneous needle aponeurotomy, reinforcing the notion that the release of mechanical constraint facilitates measurable functional recovery. These findings support the concept that digital realignment and restoration of extension directly translate into improved hand mechanics and strength.^4^

The mechanism behind the improvement in grip strength observed in our study likely involves multiple factors.^15-17^ One central hypothesis is that DD impairs optimal hand biomechanics by shortening the palmar fascia and limiting digital extension. This abnormal positioning restricts the hand’s ability to assume an optimal grip configuration, particularly during power grasp activities that rely on a fully extended metacarpal arch.^18,19^ Grip strength is a key indicator of hand function and is largely generated through the combined action of the middle finger, ring finger, and little finger.^20,21^ These 3 digits account for the majority of grip force, with the middle finger contributing the highest proportion. Flexion contractures in these digits, particularly at the PIPJ, compromise the flexor tendon arc and impair the ability to achieve a full composite grip.^22^ As a result, patients with DD often present with a measurable reduction in grip strength at baseline.^4^ By releasing the fibrotic cords responsible for fixed flexion deformity, PNA helps restore the alignment and mobility of these key gripping digits. This anatomical and biomechanical correction facilitates improved muscle activation and force transmission during grip, leading to the significant gains in grip strength observed in our study.

Additionally, prolonged contracture can lead to disuse or altered recruitment of the hand musculature.^23^ Patients may unconsciously avoid activating the full range of motion or engaging specific muscle groups due to the physical limitations imposed by the cords. The release of these contractures may therefore lead to the re-engagement of dormant neuromuscular pathways, primarily when supported by structured hand therapy.^23,24^ In our study, all patients received splinting and rehabilitation immediately following the procedure, along with education on exercises for both active and passive range of motion. This early intervention likely facilitated not only the restoration of joint mobility but also the activation of previously inhibited muscle groups.

Grip strength is a well-established biomarker of overall health and has been consistently linked to all-cause mortality in numerous population-based studies.^25,26^ Lower grip strength is associated with increased risk of cardiovascular disease, functional decline, frailty, and premature death, independent of age and comorbidities.^27^ Grip strength has been shown to outperform traditional metrics such as systolic blood pressure in predicting long-term survival outcomes in older adults.^28^ In this context, the impact of DD on grip efficiency and functional strength may have broader implications beyond localized hand dysfunction. Chronic digital contractures not only impair hand use but may subtly reduce grip capacity and contribute to functional limitation, particularly in older individuals who are already at risk of sarcopenia.^28^ By restoring digital extension and improving grip strength through interventions such as PNA, patients may not only experience better hand function but also potentially benefit from improved physical resilience and longevity.^29^ Although further longitudinal studies are needed to confirm this link, the role of hand function as a modifiable factor in healthy aging positions DD treatment within a broader framework of functional preservation and preventive care.

Percutaneous needle aponeurotomy has consistently demonstrated greater efficacy in correcting contractures of the MCPJ compared with PIPJ in DD patients. This trend has been attributed to the superficial location and relative simplicity of the cords involved at the MCPJ level, which are more accessible for percutaneous division.^30,31^ In contrast, contractures of the PIPJ involve deeper and more complex fibrous cords, as well as associated capsuloligamentous stiffness, which may limit the success of percutaneous release.^31^ However, in our study cohort, greater improvement was observed in the PIPJ compared with MCPJ. This may be explained by selection bias, where patients had more severe or earlier-stage disease involving the PIPJ. Additionally, the standardized surgical technique performed by a single experienced hand surgeon, combined with structured postoperative hand therapy, may have enhanced outcomes specifically at the PIPJ.

Another important consideration is the psychological and functional impact of contracture correction. Following successful treatment, patients often report greater confidence in using their hand, leading to increased participation in daily and occupational tasks.^32^ This increased activity may, in turn, promote strengthening and coordination of the hand musculature.^33^ Although this behavioral effect is difficult to quantify, its contribution to improved grip strength should not be underestimated.

Notably, the improvement in grip strength was achieved with minimal morbidity. Only minor skin tears and 1 case of transient hypersensitivity were noted, and all but 1 working patient returned to employment within 1 week of the procedure. Furthermore, patient-reported outcome measures at 2 months, specifically the Southampton and URAM scores, confirmed minimal disability and functional impairment. These findings underscore the safety profile of percutaneous needle aponeurotomy, which has been widely documented as a low-risk procedure suitable for an outpatient setting.

A subset of patients in this analysis was previously reported in our pilot study by Medland et al.^4^ That study involved 29 patients recruited over 5 months (February-July 2024) and served primarily as a proof-of-concept for grip strength outcomes following PNA. In contrast, this study reports on a larger cohort of 53 patients treated between February 2024 and March 2025, thereby improving statistical power and Generalizability. Key differences distinguish this work from the earlier pilot. The expanded cohort nearly doubles the number of participants and spans a 12-month recruitment window, capturing a broader clinical spectrum. Beyond grip strength at 6 weeks and 3 months, the present study incorporates validated outcome measures (Southampton and URAM scores), systematic return-to-work data, and joint-specific analyses at the MCPJ, PIPJ, and DIPJ. Methodological refinements included standardized dynamometry according to the American Society of Hand Therapists protocol, which reduced variability and improved reproducibility. The larger data set enabled subgroup analyses (eg, diabetes, occupation, and digit involvement). It demonstrated statistically significant improvements in grip strength (mean + 3.8 kg, *P* < .001), compared with the nonsignificant trend observed in the pilot. Together, these findings establish this study as a distinct extension of earlier work, providing more substantial evidence that PNA not only corrects flexion contractures but also yields measurable, clinically meaningful gains in functional hand performance.

This study has several strengths. It prospectively measured grip strength using a validated dynamometer and standardized protocol, ensuring consistency and reproducibility. A key strength lies in its focus on grip strength as an objective outcome measure, an area often neglected in previous studies that have traditionally prioritized extension deficits or recurrence rates. By systematically assessing grip strength, the study offers robust insight into real-world functional recovery following intervention. Furthermore, the incorporation of patient-reported outcome measures and documentation of return-to-work data provides a comprehensive view of the recovery process, capturing both clinical and patient-centered outcomes. However, limitations include the lack of a control group, a relatively short follow-up period, and the exclusion of additional functional assessments such as pinch strength or dexterity. Long-term studies are needed to determine whether the observed strength gains are durable and whether they translate into sustained improvements in quality of life and work capacity.

## Conclusion

Percutaneous needle aponeurotomy not only corrects digital contracture but also enhances grip strength, reflecting true functional recovery. Given its low morbidity and rapid return to activity, PNA should be considered an effective first-line option in suitable Dupuytren’s cases. Long-term studies are warranted to confirm the durability of the strength gain. Future research should explore long-term functional outcomes, durability of strength gains, and comparative effectiveness with other treatment modalities, integrating grip strength as a core functional endpoint.

## References

[1] Seth I McClure V Lim B , et al. Collagenase clostridium histolyticum versus percutaneous needle fasciotomy for Dupuytren’s disease: a systematic review and meta-analysis. Life. 2025;15(2):259. 40003669 10.3390/life15020259 PMC11856867

[2] Seth I Marcaccini G Lim K , et al. Management of Dupuytren’s disease: a multi-centric comparative analysis between experienced hand surgeons versus artificial intelligence. Diagnostics. 2025;15(5):587. 40075834 10.3390/diagnostics15050587 PMC11898831

[3] Cevik J Rajaram R Pollock M , et al. Collagenase clostridium histolyticum for Dupuytren’s disease: a comprehensive systematic review and comparative analysis against percutaneous needle aponeurotomy and limited fasciectomy. J Plast Surg Hand Surg. 2025;60:27-34. 39945007 10.2340/jphs.v60.42750

[4] Medland J Garcia N Seth I , et al. Dupuytren’s disease percutaneous needle aponeurotomy: does grip strength improve post procedure? J Clin Med. 2025;14(12):4171. 40565917 10.3390/jcm14124171 PMC12194114

[5] Ball C Pratt AL Nanchahal J. Optimal functional outcome measures for assessing treatment for Dupuytren’s disease: a systematic review and recommendations for future practice. BMC Musculoskelet Disord. 2013;14:1-11. 23575442 10.1186/1471-2474-14-131 PMC3637830

[6] Zyluk A Jagielski W. The effect of the severity of the Dupuytren’s contracture on the function of the hand before and after surgery. J Hand Surg Eur Vol. 2007;32(3):326-329. 17335947 10.1016/J.JHSB.2006.10.007

[7] Fernando JJ Fowler C Graham T , et al. Pre-operative hand therapy management of Dupuytren’s disease: a systematic review. Hand Ther. 2024;29(2):52-61. 38827652 10.1177/17589983241227162 PMC11143942

[8] Grüner JS Cai A Pingel I , et al. Prospective analysis of grip strength and load distribution after surgical treatment of common diseases of the hand with novel’s manugraphy(®) system. Arch Orthop Trauma Surg. 2023;143(10):6477-6485. doi:10.1007/s00402-023-04984-x 37486446 PMC10491509

[9] Haas-Lützenberger EM Pippich K Allgöwer K , et al. Patients with Dupuytren disease use excessive grip force when lifting and holding small objects, independent of the degree of contracture. Arch Phys Med Rehabil. 2023;104(8):1268-1273. doi:10.1016/j.apmr.2023.02.010 36893878

[10] Benson LS Williams CS Kahle M. Dupuytren’s contracture. J Am Acad Orthop Surg. 1998;6(1):24-35. 9692938 10.5435/00124635-199801000-00003

[11] Shih B Bayat A. Scientific understanding and clinical management of Dupuytren disease. Nat Rev Rheumatol. 2010;6(12):715-726. 21060335 10.1038/nrrheum.2010.180

[12] Melamed E Beutel BG Goldstein S , et al. Predictors of outcomes following fasciectomy for Dupuytren’s disease in diabetic and non-diabetic patients. J Hand Surg Asian Pac Vol. 2017;22(3):309-314. doi:10.1142/s0218810417500356 28774253

[13] Pereira A Massada M Sousa R , et al. Percutaneous needle fasciotomy in Dupuytren’s contracture: is it a viable technique? Acta Orthop Belg. 2012;78(1):30-34. 22523924

[14] Hurst LC Badalamente MA Hentz VR , et al. Injectable collagenase clostridium histolyticum for Dupuytren’s contracture. N Engl J Med. 2009;361(10):968-979. doi:10.1056/NEJMoa0810866 19726771

[15] Maurissen JP Marable BR Andrus AK , et al. Factors affecting grip strength testing. Neurotoxicol Teratol. 2003;25(5):543-553. 12972067 10.1016/s0892-0362(03)00073-4

[16] Turesson C. The role of hand therapy in Dupuytren disease. Hand Clin. 2018;34(3):395-401. 30012299 10.1016/j.hcl.2018.03.008

[17] Rantanen T Volpato S Ferrucci L , et al. Handgrip strength and cause-specific and total mortality in older disabled women: exploring the mechanism. J Am Geriatr Soc. 2003;51(5):636-641. 12752838 10.1034/j.1600-0579.2003.00207.x

[18] Rath S. Hand kinematics: application in clinical practice. Indian J Plast Surg. 2011;44(2):178-185. 22022027 10.4103/0970-0358.85338 PMC3193629

[19] DeMott L Flinn SR . Functional anatomy. In: Wietlisbach CM , ed. Cooper’s Fundamentals of Hand Therapy: Clinical Reasoning and Treatment Guidelines for Common Diagnoses of the Upper Extremity. Mosby; 2019:21-45.

[20] Cha SM Shin HD Kim KC , et al. Comparison of grip strength among 6 grip methods. J Hand Surg Am. 2014;39(11):2277-2284. 25085045 10.1016/j.jhsa.2014.06.121

[21] Kong YK Kim DM. The relationship between hand anthropometrics, total grip strength and individual finger force for various handle shapes. Int J Occup Saf Ergon. 2015;21(2):187-192. 26323777 10.1080/10803548.2015.1029726

[22] Kuran B. Functional assessment in hand with flexor and extensor tendon injuries. In: Duruöz M , ed. Hand Function: A Practical Guide to Assessment. Springer; 2019:109-124.

[23] Wang F Zhang QB Zhou Y , et al. The mechanisms and treatments of muscular pathological changes in immobilization-induced joint contracture: a literature review. Chin J Traumatol. 2019;22(2):93-98. 30928194 10.1016/j.cjtee.2019.02.001 PMC6488749

[24] Appell HJ. Muscular atrophy following immobilisation: a review. Sports Med. 1990;10(1):42-58. 2197699 10.2165/00007256-199010010-00005

[25] Vaishya R Misra A Vaish A , et al. Hand grip strength as a proposed new vital sign of health: a narrative review of evidences. J Health Popul Nutr. 2024;43(1):7. 38195493 10.1186/s41043-024-00500-y PMC10777545

[26] Bohannon RW. Grip strength: an indispensable biomarker for older adults. Clin Interv Aging. 2019;14:1681-1691. 31631989 10.2147/CIA.S194543 PMC6778477

[27] Reeve TE 4th Ur R Craven TE , et al. Grip strength measurement for frailty assessment in patients with vascular disease and associations with comorbidity, cardiac risk, and sarcopenia. J Vasc Surg. 2018;67(5):1512-1520. 29276105 10.1016/j.jvs.2017.08.078

[28] Laukkanen JA Voutilainen A Kurl S , et al. Handgrip strength is inversely associated with fatal cardiovascular and all-cause mortality events. Ann Med. 2020;52(3-4):109-119. 32223654 10.1080/07853890.2020.1748220 PMC7877981

[29] Rantanen T Guralnik JM Foley D , et al. Midlife hand grip strength as a predictor of old age disability. JAMA. 1999;281(6):558-560. 10022113 10.1001/jama.281.6.558

[30] Yoon AP Kane RL Hutton DW , et al. Cost-effectiveness of recurrent Dupuytren contracture treatment. JAMA Netw Open. 2020;3(10):e2019861. doi:10.1001/jamanetworkopen.2020.19861 PMC7545302 33030553

[31] Mella JR Guo L Hung V. Dupuytren’s contracture: an evidence based review. Ann Plast Surg. 2018;81(6S suppl 1):S97-S101. 10.1097/SAP.0000000000001607 30161050

[32] Sanjuan-Cervero R Gomez-Herrero D Poquet-Jornet JE , et al. A comparison of patient-reported outcome measures for Dupuytren disease: a prospective view. J Plast Reconstr Aesthet Surg. 2022;75(10):3774-3781. 36028430 10.1016/j.bjps.2022.06.024

[33] Mon Y Seth I Medland J , et al. Effectiveness of splinting after percutaneous needle fasciotomy for Dupuytren’s contracture. J Hand Surg Eur Vol. Published online June 17, 2024. doi:10.1177/17531934251350463 40525787

